# Phenotype Algorithms to Identify Hidradenitis Suppurativa Using Real-World Data: Development and Validation Study

**DOI:** 10.2196/38783

**Published:** 2022-11-30

**Authors:** Jill Hardin, Gayle Murray, Joel Swerdel

**Affiliations:** 1 Janssen Research and Development Titusville, NJ United States; 2 Observational Health Data Sciences and Informatics New York, NY United States

**Keywords:** dermatology, hidradenitis suppurativa, medical dermatology, observational data, phenotype, inflammation, skin disease, epidemiology, algorithm

## Abstract

**Background:**

Hidradenitis suppurativa (HS) is a potentially debilitating, chronic, recurring inflammatory disease. Observational databases provide opportunities to study the epidemiology of HS.

**Objective:**

This study’s objective was to develop phenotype algorithms for HS suitable for epidemiological studies based on a network of observational databases.

**Methods:**

A data-driven approach was used to develop 4 HS algorithms. A literature search identified prior HS algorithms. Standardized databases from the Observational Medical Outcomes Partnership (n=9) were used to develop 2 incident and 2 prevalent HS phenotype algorithms. Two open-source diagnostic tools, CohortDiagnostics and PheValuator, were used to evaluate and generate phenotype performance metric estimates, including sensitivity, specificity, positive predictive value (PPV), and negative predictive value.

**Results:**

We developed 2 prevalent and 2 incident HS algorithms. Validation showed that PPV estimates were highest (mean 86%) for the prevalent HS algorithm requiring at least two HS diagnosis codes. Sensitivity estimates were highest (mean 58%) for the prevalent HS algorithm requiring at least one HS code.

**Conclusions:**

This study illustrates the evaluation process and provides performance metrics for 2 incident and 2 prevalent HS algorithms across 9 observational databases. The use of a rigorous data-driven approach applied to a large number of databases provides confidence that the HS algorithms can correctly identify HS subjects.

## Introduction

Hidradenitis suppurativa (HS) is a chronic, recurring inflammatory disease of the skin. Clinically, subjects have nodules, draining skin tunnels (ie, sinus tracts), abscesses, and bands of severe scar formation in the intertriginous skin areas, such as the axillary, groin, perianal, perineal, and inframammary regions [1]. Patients with HS suffer from metabolic, psychiatric, and autoimmune disorders [2].

The use of real-world evidence from observational data is valuable for studying the epidemiology, clinical manifestations, and real-world experience of patients with HS. A critical step in using observational data for the study of HS is the development of accurate phenotype algorithms (PAs). A PA is the translation of the case definition of a health condition or phenotype into an executable algorithm based on clinical data elements in a database [3]. Several studies have investigated HS using health care claims, electronic medical records, patient care, and hospitalization databases and have been conducted using data from the United States, Germany, Finland, Taiwan, Korea, England, Canada, and Denmark [2,4-32]. These studies have focused on a range of topics in patients with HS, including the incidence and prevalence of HS in different populations and the associations between HS and autoimmune disorders. Only 5 studies have provided phenotype validation metrics [9,10,16,29,30]; 2 used hospital data [16,29], 4 used a single phenotype requiring at least one code for HS from the International Classification of Diseases, Ninth Revision (ICD-9) [9,10,29,30], and 1 evaluated several phenotypes [16].

The objectives of this study were to develop HS PAs, evaluate their performance, and characterize the resultant HS phenotypes across a network of 9 US and non-US observational databases. This study used a data-driven framework and developed HS PAs for use in observational databases.

## Methods

### Overview

A literature search was conducted to identify studies that describe the codes and logic used to identify HS patients in observational databases. This literature search identified 30 articles, which provided a set of diagnosis codes for the identification of HS across vocabularies, including the ICD-9, the International Classification of Diseases, Tenth Revision (ICD-10), and Read codes. Five of the 30 articles included validation metrics. Our study utilized the Systemized Nomenclature of Medicine (SNOMED) vocabulary to develop the codes. The vocabulary and diagnostic codes used in the published studies and the SNOMED terms are presented in Multimedia Appendix 1. The Observational Health Data Sciences and Informatics (OHDSI) open-source Atlas tool [33] was used to create the HS PAs.

The observational databases used in this study were not created specifically to study HS. The observational data were obtained in the delivery of health care or for administrative or billing purposes in electronic format. A network of 9 observational databases (4 administrative claims databases from the United States, 1 from Japan, 1 from France, 1 from Germany, and 1 from Australia; and 1 US electronic health record [EHR] database; Table 1) were used to develop the PAs. The 9 databases were a mix of administrative insurance claims, EHRs, and general practitioner databases. Descriptions and details of each database are shown in Table 2. The databases were transformed to the Observational Medical Outcomes Partnership (OMOP) Common Data Model (version 5.3.1) [34] so the PAs could be consistently applied across databases.

**Figure 1:**
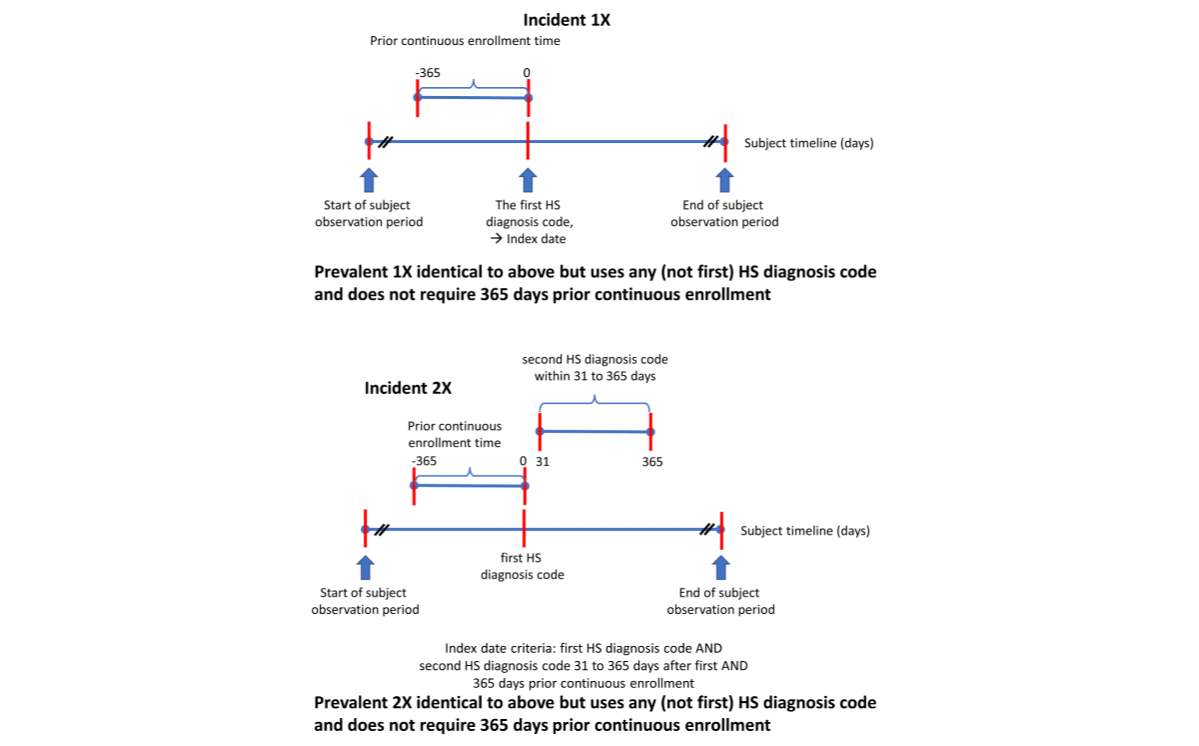
Schematics of phenotype algorithms for Hidradenitis suppurativa (HS).

Four HS PAs were developed and evaluated in subjects of all ages [35] (Figure 1). The PA “incident 1x” used the first diagnosis code for HS in a subject’s history and required 365 days of prior continuous enrollment (CE) time to qualify for entry into the HS cohort. The date a subject met both criteria was the subject’s index date. The PA “incident 2x” used the first diagnosis code for HS in a subject’s history and required both a second HS diagnosis code within 31 to 365 days and 365 days of prior CE time. The date a subject met all 3 criteria became the subject’s index date. The prevalent PAs (“prevalent 1x” and “prevalent 2x”) were identical to the corresponding incident versions, except that the first HS diagnosis code was not required to be the first time an HS code occurred in a subject’s history, nor was there a requirement for 365 days prior CE.

The OHDSI CohortDiagnostics tool [36] allowed for evaluation and comparison of PAs at a cohort level, providing overall counts, incidence over time, the diagnosis code that allowed the subject into the cohort, cohort overlap, and temporal characterization.

Use of the PheValuator [37] method provided performance metrics, including the sensitivity, specificity, positive predictive value (PPV), and negative predictive value (NPV) associated with each PA. PheValuator is a machine learning–based method of assessing PAs. It constructs a predictive model for the disease and calculates the predictive value of having the disease for each subject using the model. Using PheValuator, performance indices of an algorithm are calculated without reviewing medical charts. While algorithm validation results from chart review are considered the “gold standard,” we have compared the results from PheValuator with prior studies using chart review and found excellent agreement between the 2 methods [38]. Four additional PAs from Kim et al [16] were evaluated for comparison.

Computer code for PheValuator and CohortDiagnostics and the JSON files for the PAs are available on the authors’ website [39].

**Table 1:** Description of databases used in the study.

Name	Years	Country	Data type	Clinical visits included	Subjects, n (millions)	Age at first observation, average (years)	Female subjects, %	Length of follow-up, median (years)
IBM MarketScan Commercial Claims and Encounters	2000-2021	United States	Insurance claims	Inpatient/outpatient	157	31	51	1.56
IBM MarketScan Multi-State Medicaid	2006-2020	United States	Insurance claims	Inpatient/outpatient	31	23	56	1.52
IBM MarketScan Medicare Supplemental	2000-2021	United States	Insurance claims	Inpatient/outpatient	10	71	55	2.46
Optum’s de-identified Clinformatics Data Mart Database	2007-2021	United States	Insurance claims	Inpatient/outpatient	71	37	51	1.48
Optum Electronic Health Records	2007-2021	United States	Electronic health records	Inpatient/outpatient	99	37	53	2.63
Japan Medical Data Center	2000-2021	Japan	Insurance claims	Inpatient/outpatient	12	31	49	3.29
IQVIA Disease Analyzer–France	2016-2021	France	General practitioner data	Outpatient	4	37	52	0.9
IQVIA Disease Analyzer–Germany	2011-2021	Germany	General practitioner data with supplemental data from participating specialists	Outpatient	31	43	56	0.5
IQVIA Australian Longitudinal Patient Data	1996-2020	Australia	General practitioner data	Outpatient	5	37	22^a^	0.5

^a^59% of subjects did not have a designated sex in this study.

### Ethics Approval

The use of the IBM and Clinformatics databases was reviewed by the New England Institutional Review Board and was determined to be exempt from broad approval, as this project did not involve human subject research. Patient consent for publication was not required. All patients in the databases were deidentified, and the identities of data contributors were removed.

## Results

**Figure 2:**
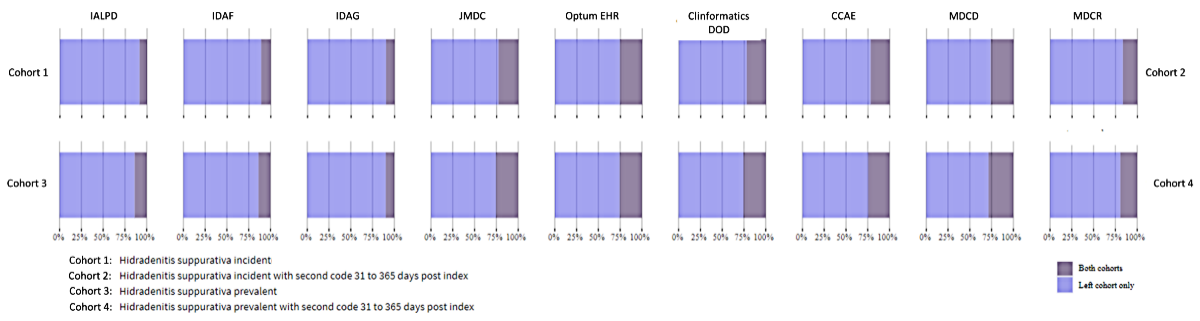
Graphical depiction of the overlap in subjects between the 2 incidence cohorts and the 2 prevalence cohorts. CCAE: IBM MarketScan Commercial Claims and Encounters; Clinformatics DOD: Optum’s de-identified Clinformatics Data Mart Database; IALPD: IQVIA Australian Longitudinal Patient Data; IDAF: IQVIA Disease Analyzer–France; IDAG: IQVIA Disease Analyzer–Germany; JMDC: Japan Medical Data Center; MDCD: IBM MarketScan Multi-State Medicaid; MDCR: IBM MarketScan Medicare Supplemental; Optum EHR: Optum Electronic Health Records.

We examined cohort characteristics of the PAs. These characteristics may be viewed interactively online [40]. The number of subjects ranged from 81 in the IQVIA Australian Longitudinal Patient Data (IALPD) database to 170,149 in the IBM MarketScan Commercial Claims and Encounters (CCAE) database for the incident 1x cohort. These numbers were as expected based on the relative sizes of the databases, indicating that all codes used were appropriate for each database. The counts were much higher in the US databases compared to the non-US databases. The reduction in the number of subjects in the incident 1x PA compared to the incident 2x PA ranged from about 90% in the IALPD, IQVIA Disease Analyzer–France (IDAF), and IQVIA Disease Analyzer–Germany (IDAG) databases to about 73% in the IBM MarketScan Multi-State Medicaid (MDCD) database. The incident 1x PA identified a higher proportion of female subjects compared to male subjects: 51% in the Japan Medical Data Center (JMDC) database and 81% in the MDCD database; the incident 2x PA identified a lower proportion of female subjects in the JMDC database (46%) but a higher proportion in all other databases, ranging from 53% for the IDAG database to 82% for the MDCD database. The overlap in subjects between the incident PAs for each database is shown in Figure 2. The incident 2x PA is a subset of the incident 1x PA.

**Figure 3:**
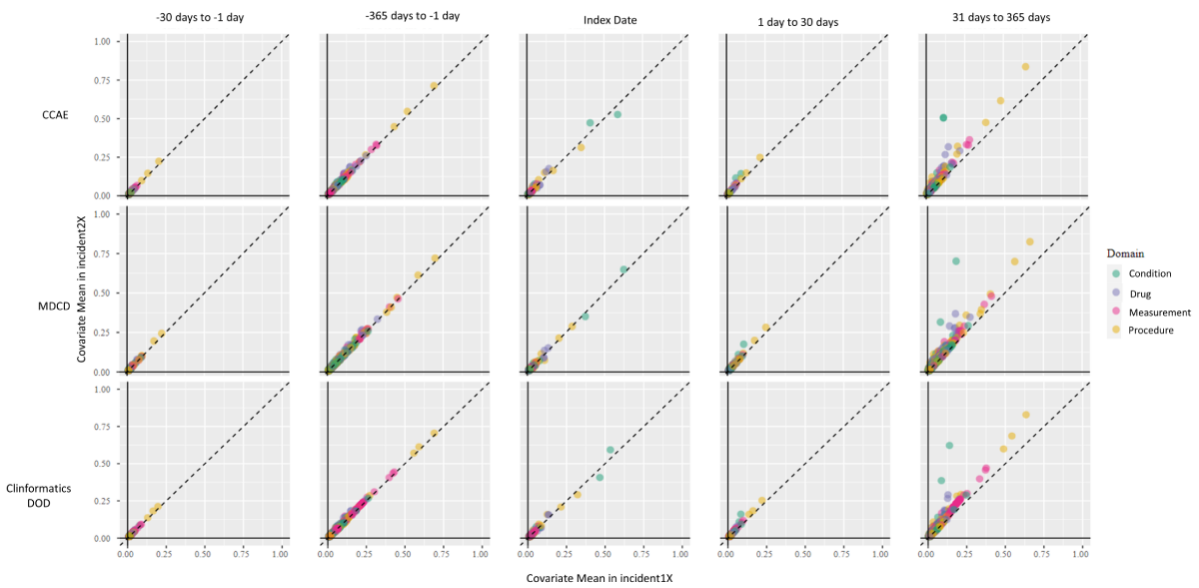
Comparison of the proportion of subjects in the incident 1x cohort and the incident 2x cohort for 3 selected data sets with different demographic characteristics. Points closer to the diagonal indicate similar proportions between the comparators; points farther from the diagonal indicate more disparate proportions. CCAE: IBM MarketScan Commercial Claims and Encounters; Clinformatics DOD: Optum’s de-identified Clinformatics Data Mart Database; MDCD: IBM MarketScan Multi-State Medicaid.

A comparison of standardized differences between the incident 1x and the incident 2x cohorts for 3 data sets across 5 different time frames is shown in Figure 3. Differences in the standardized difference of the mean greater than 0.1 are considered imbalanced [41]. Points closer to the diagonal indicate similar proportions between cohorts; points farther from the diagonal indicate more disparate proportions. The plots compare the diagnosed conditions, prescribed drugs, laboratory measurements, and clinical procedures of the subjects in the incident 1x and incident 2x PA cohorts and illustrate the population differences. The CCAE database showed disparities between the 2 algorithms in the period 31 to 365 days after the index date. Some differences arose from higher proportions of diagnosis codes for HS (50% for incident 2x vs 11% for incident 1x, standard mean difference [SMD] 0.66) and prescriptions for clindamycin (32% for incident 2x vs 14% for incident 1x, SMD 0.3). There were also differences in the MDCD database population, with more subjects of a lower socioeconomic status. The MDCD database also showed differences in diagnosis codes for HS (70% for incident 2x vs 18% for incident 1x, SMD 0.86) and prescriptions for clindamycin (37% for incident 2x vs 13% for incident 1x, SMD 0.31). The Optum’s de-identified Clinformatics Data Mart Database (Clinformatics DOD) data set showed differences in proportions between the 2 cohorts for diagnosis codes for HS (62% for incident 2x vs 14% for incident 1x, SMD 0.81) and prescriptions for clindamycin (29% for incident 2x vs 13% for incident 1x, SMD 0.29). The relative proportions between the 2 cohorts for the majority of the characteristics in the CCAE, MDCD, and Clinformatics DOD databases showed similar proportions between the cohorts.

We examined the incident 2x algorithm for subject characteristics across the databases. We identified a higher proportion of female subjects with HS compared to male subjects. The largest disproportionality was in the MDCD database, in which 82% of the subjects were female. The JMDC database had the lowest disproportionality by sex, with 45% female subjects. An outpatient visit was the most common type of clinical visit for the first diagnosis of HS. Less than 5% of first diagnoses were made during an emergency room visit, with the exception of the MDCD database, for which the proportion was 10%. Examination of the index codes or diagnosis codes that allowed subjects into cohorts showed that the most prevalent code was the diagnosis code of “hidradenitis suppurativa” (SNOMED code 4241223; ICD-10 L73.2) in all databases except the CCAE database, in which the most prevalent code was a diagnosis code of “hidradenitis” (SNOMED code 434119; ICD-9 705.83).

Incidence rates for HS (for the incident 2x algorithm) from 2015 to 2020 differed between databases. The MDCD database had the highest rate at 23 per 100,000 person-years. The rates in the CCAE, Clinformatics DOD, and Optum EHR databases were approximately 12 per 100,000 person-years. Rates in the IDAG and IDAF databases and the JMDC and the IBM MarketScan Medicare Supplemental Database (MDCR) databases were 1 per 100,000 person-years. The rate in the IALPD database was undetectable, likely due to the small sample size. The incidence rates peaked in subjects in the 20- to 29-year-old age group. The incidence rates in the 30- to 39-year-old age group in the MDCD and IDAG databases were higher than in the older age groups but were similar to the 20- to 29-year-old age group. Incidence rates in female subjects were generally higher than in male subjects and were highest in the MDCD database at 24 per 100,000 person-years, followed by 11 per 100,000 person-years in the CCAE, Clinformatics DOD, and Optum EHR databases and 1 per 100,000 person-years in the IDAF database. The rate in female subjects was equal to the rate in male subjects in the MDCR database at 2 per 100,000 person-years.

Performance characteristics for the HS phenotypes assessed using the PheValuator method are presented in Table 2. Due to low subject counts, calculation of performance characteristics for the IDAG, IDAF, IALPD, and JMDC databases was not possible. The mean PPVs were higher in all databases for the PAs requiring a second diagnostic HS code in the 31 to 365 days after the index date. The mean PPVs for the 2 PAs that required a second code was 88% (incident) and 86% (prevalent). This was reduced to 62% (incident) and 59% (prevalent) when only a single diagnosis code for HS was required. The highest sensitivity estimates were in the 2 prevalent cohorts. The sensitivity for the 2 prevalent algorithms was 58% (single code required) and 25% (2 codes required). This decreased to 32% (single code required) and 12% (2 codes required) in the incident cohorts. The estimates for mean PPV for the Kim et al [16] PAs increased with the increase in number of HS diagnosis codes, ranging from 59% (2 codes) to 84% (5 codes). Our results showed a similar trend, but PPV was lower than reported by Kim et al (81% including subjects with 2 HS codes and 97% including subjects with >5 codes).

**Table 2:** Performance characteristics of the hidradenitis suppurativa phenotypes based on the PheValuator methodology.

Phenotype algorithm/database		Sensitivity (95% CI)		PPV^a^ (95% CI)		Specificity (95% CI)		NPV^b^ (95% CI)
**Hidradenitis suppurativa incidence**								
	IBM MarketScan Commercial Database	0.380 (0.367-0.393)	0.599 (0.582-0.615)		0.999 (0.999-0.999)		0.998 (0.998-0.998)	
	Optum’s de-identified Clinformatics Data Mart Database	0.369 (0.358-0.380)	0.603 (0.589-0.617)		0.999 (0.999-0.999)		0.998 (0.997-0.998)	
	IBM MarketScan Multi-State Medicaid Database	0.311 (0.306-0.317)	0.676 (0.668-0.685)		0.998 (0.998-0.998)		0.990 (0.990-0.990)	
	IBM MarketScan Medicare Supplemental Database	0.298 (0.277-0.319)	0.444 (0.417-0.472)		1.000 (1.000-1.000)		0.999 (0.999-0.999)	
	Optum’s de-identified Electronic Health Record dataset	0.279 (0.269-0.289)	0.777 (0.761-0.793)		1.000 (1.000-1.000)		0.997 (0.997-0.997)	
**Hidradenitis suppurativa incidence with second diagnosis 31 to 365 days after index date**								
	IBM MarketScan Commercial Database	0.151 (0.142-0.161)	0.890 (0.868-0.909)		1.000 (1.000-1.000)		0.998 (0.998-0.998)	
	Optum’s de-identified Clinformatics Data Mart Database	0.133 (0.126-0.141)	0.882 (0.862-0.900)		1.000 (1.000-1.000)		0.997 (0.996-0.997)	
	IBM MarketScan Multi-State Medicaid Database	0.115 (0.112-0.119)	0.874 (0.862-0.885)		1.000 (1.000-1.000)		0.987 (0.987-0.987)	
	IBM MarketScan Medicare Supplemental Database	0.109 (0.095-0.123)	0.830 (0.778-0.874)		1.000 (1.000-1.000)		0.999 (0.999-0.999)	
	Optum de-identified Electronic Health Record dataset	0.109 (0.102-0.116)	0.948 (0.931-0.962)		1.000 (1.000-1.000)		0.997 (0.996-0.997)	
**Hidradenitis suppurativa prevalence**								
	IBM MarketScan Commercial Database	0.541 (0.531-0.551)	0.649 (0.639-0.660)		0.999 (0.999-0.999)		0.998 (0.998-0.998)	
	Optum’s de-identified Clinformatics Data Mart Database	0.666 (0.655-0.677)	0.602 (0.591-0.613)		0.998 (0.998-0.998)		0.999 (0.999-0.999)	
	IBM MarketScan Multi-State Medicaid Database	0.664 (0.658-0.670)	0.628 (0.621-0.634)		0.995 (0.995-0.995)		0.996 (0.996-0.996)	
	IBM MarketScan Medicare Supplemental Database	0.442 (0.422-0.462)	0.355 (0.338-0.373)		0.999 (0.999-0.999)		0.999 (0.999-0.999)	
	Optum de-identified Electronic Health Record dataset	0.632 (0.618-0.647)	0.754 (0.739-0.768)		1.000 (1.000-1.000)		0.999 (0.999-0.999)	
**Hidradenitis suppurativa prevalence with second diagnosis 31 to 365 days after index date**								
	IBM MarketScan Commercial Database	0.296 (0.285-0.307)	0.874 (0.860-0.887)		1.000 (1.000-1.000)		0.997 (0.997-0.998)	
	Optum’s de-identified Clinformatics Data Mart Database	0.233 (0.220-0.246)	0.937 (0.920-0.951)		1.000 (1.000-1.000)		0.998 (0.998-0.998)	
	IBM MarketScan Multi-State Medicaid Database	0.219 (0.203-0.236)	0.732 (0.699-0.764)		1.000 (1.000-1.000)		0.999 (0.999-0.999)	
	IBM MarketScan Medicare Supplemental Database	0.288 (0.282-0.294)	0.859 (0.851-0.867)		0.999 (0.999-0.999)		0.992 (0.992-0.992)	
	Optum de-identified Electronic Health Record dataset	0.231 (0.222-0.239)	0.912 (0.900-0.923)		1.000 (1.000-1.000)		0.996 (0.996-0.996)	

^a^PPV: positive predictive value.

^b^NPV: negative predictive value.

## Discussion

### Principal Findings

This study sought to develop and determine the accuracy of 4 HS PAs. The 4 PAs included 2 for incidence and 2 for prevalence, with one in each group having high sensitivity and specificity. Use of the PheValuator method allowed for estimation of sensitivity, specificity, PPV, and NPV without manual chart review. While both the incident and prevalent PAs were useful for the exploration of HS in observational databases, the PAs with definitions requiring just a single HS diagnosis code had lower specificity and higher sensitivity than the definitions requiring 2 codes, which had higher specificity and lower sensitivity. Thus, the choice of which algorithm to use is dependent on the research question being explored. For example, the use of a more sensitive algorithm would be applicable for safety studies, in which the PA is used to determine HS outcomes and missed identification of possible cases is problematic, whereas the use of a PA with higher specificity would be useful for treatment comparison studies, in which the goal is to ensure that all subjects exposed to a treatment have a high probability of having HS.

A few studies have included validation metrics for HS algorithms for observational databases [9,10,16,29,30]. Kim et al [16] used data available from the Massachusetts General Hospital and reported an increase in PPV with an increasing number of HS diagnosis codes (81% for 2 codes vs 97% for 5 codes). Our study replicated the Kim et al cohorts and found an increase in PPV with the use of 5 or more diagnosis codes compared to the use of at least two HS diagnosis codes (mean 84% for >5 codes vs mean 59% for 2 codes) that was similar to, albeit lower than, the published results. In general, our study found higher PPVs compared to studies that used a single HS diagnosis code [9,10,29,30]. The majority of subjects identified in our study were female, which is similar to findings from other studies [5,6,9,16,31]. A US study that used a cross-sectional design and a large electronic medical records database found an overall prevalence of 24.8% for type 2 diabetes, 71.6% for obesity, and 39.9% for hyperlipidemia among HS subjects [8]. Our study, when restricted to US data and examining covariates 365 days prior to and including the index date, identified type 2 diabetes in 26.5%, obesity in 19.6%, and hyperlipidemia in 26.5% of incident 1x HS subjects. The cross-sectional study was restricted to subjects aged 18 years or older, while our study included all ages, which may help in interpreting the decreased proportion of hyperlipidemia observed in our results. It has been reported that administrative databases underreport obesity as a diagnosis and are not an optimal data source for obesity prevalence [42]. This may support our finding of a lower prevalence of obesity compared to the findings of the cross-sectional study.

Strengths of our study include the use of a rigorous, data-driven approach for generating and evaluating the HS phenotypes across a data network that included 9 databases covering US and non-US countries. Network-based phenotype evaluations greatly strengthen the knowledge base for a given algorithm, because they allow the assessment of the consistency of findings across data types, geographic locations, and time periods. When concordant trends emerge, it increases confidence that the observations are the effect of the PA itself rather than an artifact of a particular data source. The PAs were analyzed using multiple approaches, providing ancillary verification of decisions made in determining the cohort logic. Our study includes several study artifacts, including JSON files for the PAs, computer code, and results for all the analyzed PAs, providing transparency in our interpretation of the results.

There were also several limitations to our study. We used administrative data sets primarily maintained for insurance billing, which are well-known to have significant deficits, including coding inaccuracies [43]. In addition, the estimation of performance characteristics using the PheValuator methodology was dependent on the quality of the data in the data set, which can vary substantially [37]. The algorithm validation was performed using a method involving predictive modeling of HS rather than case reviews. Results from PheValuator have been compared to results from previously published validation studies and have demonstrated excellent agreement [38]. This method does have the advantage of using multiple databases to provide a full set of performance metrics, including sensitivity and specificity, which are rarely provided in validation studies using case reviews [37]. The generalizability of our findings to uninsured populations is uncertain, given the insured population that was observed in this study. In the incident PA that defined HS with only a single diagnosis code, it was not possible to determine if any of these were “rule-out” diagnoses. The algorithms presented in this study use codes specific to HS; therefore, jurisdictions and practices that do not use these specific codes and instead use codes for “abscess” or “cyst” would be unable to operationalize these PAs. The study period used for evaluation of the HS algorithms includes the year (2015) when the drug Humira was introduced to treat HS [44]. Education on HS increased, and physicians became more likely to use diagnosis codes specifically indicating HS in observational data. Therefore, to avoid temporal bias, researchers should avoid use of these algorithms in data from prior to 2015.

### Conclusions

This study developed and evaluated 4 HS PAs using a rigorous, data-driven approach and generated phenotype performance metrics including sensitivity, specificity, PPV, and NPV. Based on the analyses, we recommend that PAs requiring a single HS diagnosis code be used in studies requiring high sensitivity, while studies requiring high specificity should use PAs requiring 2 HS diagnosis codes. These algorithms will enable researchers to use large observational databases to research HS, which has a high burden of disease. There is a need for better evidence, as currently there are clinical knowledge gaps for HS that observational data is well suited to address.

## Appendix

Multimedia Appendix 1 Diagnostic codes.

## Abbreviations

CCAE: IBM MarketScan Commercial Claims and Encounters
CE: continuous enrollment
Clinformatics DOD: Optum’s de-identified Clinformatics Data Mart Database
EHR: electronic health record
HS: hidradenitis suppurativa
IALPD: IQVIA Australian Longitudinal Patient Data
ICD-9: International Classification of Diseases, Ninth Revision
ICD-10: International Classification of Diseases, Tenth Revision
IDAF: IQVIA Disease Analyzer–France
IDAG: IQVIA Disease Analyzer–Germany
JMDC: Japan Medical Data Center
MDCD: IBM MarketScan Multi-State Medicaid
MDCR: IBM MarketScan Medicare Supplemental
NPV: negative predictive value
OHDSI: Observational Health Data Sciences and Informatics
OMOP: Observational Medical Outcomes Partnership
PA: phenotype algorithm
PPV: positive predictive value
SMD: standard mean difference
SNOMED: Systemized Nomenclature of Medicine

## Data Availability

The data used for this study are proprietary and only available through a licensing data-use agreement process. This process ensures that confidentiality of the data contributors is maintained and that the data are used appropriately. The MarketScan Research Database can be licensed by researchers.
