# Supplementary material for: Phenotype Algorithms to Identify Hidradenitis Suppurativa Using Real-World Data: Development and Validation Study
Source: JMIR Dermatol. 2022 Nov 30;5(4):e38783. doi: 10.2196/38783 (PMC10334943; doi:10.2196/38783)
Supplement: Multimedia Appendix 1 [file derma_v5i4e38783_app1.docx]

Journal Name: JMIR Dermatology

Title: Development of Phenotype Algorithms to Identify Hidradenitis Suppurativa Using Real World Data

Authors: Jill Hardin PhD MS MBA^1,2^; Gayle Murray MSW^1^, Joel Swerdel PhD MS MPH^1,2^

^1^Janssen Research and Development, Raritan, NJ, USA; ^2^Observational Health Data Sciences and

Informatics (OHDSI), New York, NY

Corresponding author:

Jill Hardin

Department of Epidemiology

Janssen Research & Development, LLC

1125 Trenton-Harbourton Road

Titusville, NJ 08560

Email: jhardi10@its.jnj.com

| Vocabulary | Code | Description |
| --- | --- | --- |
| ICD-9 | 705.83 | Hidradenitis |
| ICD-9 | 7058C | Hidradenitis |
|  |  |  |
| ICD-10 | L729 | Follicular cyst of the skin and subcutaneous tissue, unspecified. |
| ICD-10 | L73.2 | Hidradenitis suppurativa |
|  |  |  |
| OHIP billing codes | R059 | other lesions - Inguinal, perineal or axillary skin and sweat glands for hyperhydrosis and/or hydradenitis - unilateral |
| OHIP billing codes | R060 | Inguinal, perineal or axillary skin and sweat glands for hyperhydrosis and/or hydradenitis - with skin graft(s) or rotation flap(s) |
|  |  |  |
| Read code | M25y111 | Hidradenitis suppurativa |
|  |  |  |
| SNOMED Codes | 434119 | Hidradenitis |
|  | 4241223 | Hidradenitis suppurativa |
|  | 4300122 | Axillary hidradenitis suppurativa |
|  | 4300123 | Vulval hidradenitis suppurativa |
|  | 4087429 | Hidradenitis suppurativa of anus |
|  | 4300121 | Retro-auricular cysts |
|  | 4297360 | Follicular occlusion tetrad - hidradenitis, acne conglobata, dissecting cellulitis, pilonidal sinus |
|  | 4300792 | Follicular occlusion triad - hidradenitis, acne conglobata, dissecting cellulitis of scalp |
|  | 4298741 | Anogenital hidradenitis suppurativa |
|  |  |  |
| ICD - International Classification of Diseases; SNOMED - Systemized Nomenclature of Medicine | | |
